# Patient Satisfaction With Dental Services Provided by Dental Interns at Gulf Medical University Dental Training Hospital

**DOI:** 10.7759/cureus.86522

**Published:** 2025-06-22

**Authors:** Lara Kanjee, Marwah Abdullah, Noor Alyasiri, Bassem Mohamed

**Affiliations:** 1 General Dentistry, Gulf Medical University, Ajman, ARE; 2 Restorative Dental Sciences, Gulf Medical University, Ajman, ARE

**Keywords:** dental, endodontics, extraction, filling, patient satisfaction, prosthodontics, restoration

## Abstract

Background: Patient satisfaction is an important factor in the success and quality assurance of healthcare facilities, including dental educational hospitals.

Objective: This study aims to assess patient satisfaction with dental services provided by dental interns at Thumbay Dental Training Hospital (TDTH), Ajman.

Materials and methods: A total of 251 participants were randomly selected. A questionnaire was used to measure patient satisfaction with clinical dental services performed by dental interns. Patients were asked about their experiences with dental treatments, preoperative and postoperative pain, and their satisfaction with both the dental intern and the provided treatment. Descriptive statistical analysis and the Chi-square test were used to analyze data. The level of significance was set at p<0.05 with a 95% CI.

Results: Patients treated by dental interns at TDTH were significantly satisfied (99.2%) with the dental treatment they received (p<0.001). The most frequently performed dental treatment that was provided by the interns was composite restorations (82/295; 27.8%), followed by endodontic treatment (63/295; 21.4%), and (60/295; 20%) dental extractions. Patients seeking pain relief were significantly more satisfied (66.1%) than those who came for routine care (33.9%) (p<0.001).

Conclusion: The findings indicate that the quality of care provided by dental interns exceeds patient expectations, particularly among those seeking pain relief.

## Introduction

Patient satisfaction is considered one of the most important variables that determine and outline the success of any healthcare facility. There is a wide agreement that it is almost impossible to provide high-quality treatment without measuring the patient's satisfaction [1-3]. However, satisfaction may vary depending on each individual's personal definition of quality [4]. Most studies on patient satisfaction in dental healthcare emphasize the importance of gathering patient feedback to enhance the effectiveness of dental facilities and ensure that patient needs and expectations are met during dental visits [1-3,5-9].

The internship year is an important phase for dental students, marking the transformative journey from academia to professional practice. It bridges the gap between theoretical knowledge and real-world application, allowing interns to refine their clinical skills, build confidence, and develop critical decision-making abilities. This phase allows for independence and adaptability as students navigate diverse cases and interact with patients under supervision. It also implies professional ethics, teamwork, and patient communication skills, setting the foundation for a successful career in dentistry and the first confident step into the responsibilities of being a professional dentist [10,11].

Educational hospitals like Thumbay Dental Training Hospital (TDTH), Gulf Medical University, play a crucial role in acknowledging and assessing patient satisfaction, as they provide interns with a supervised environment to gain hands-on experience across multiple specialties. By actively seeking and evaluating patient feedback, these institutions help interns develop their clinical abilities, professionalism, and patient-centered approach. This process not only enhances the quality of care but also equips future dentists with essential skills and insights needed to excel in their careers. Despite the importance of patient satisfaction in dental care, there is a notable gap in research within educational hospitals. To our knowledge, no studies have been conducted in this hospital to measure patient satisfaction with services provided by interns. This highlights the significance of our study, as it aims to address this gap and contribute valuable insights that can improve the quality of care and enhance the training experience for dental interns.

## Materials and methods

Ethical approval

This study was approved by the Institutional Review Board of Gulf Medical University, Ajman, UAE (IRB-COD-STD-57-NOV-2023). The study was conducted in full accordance with the World Medical Association Declaration of Helsinki.

Study design

**Figure 1:**
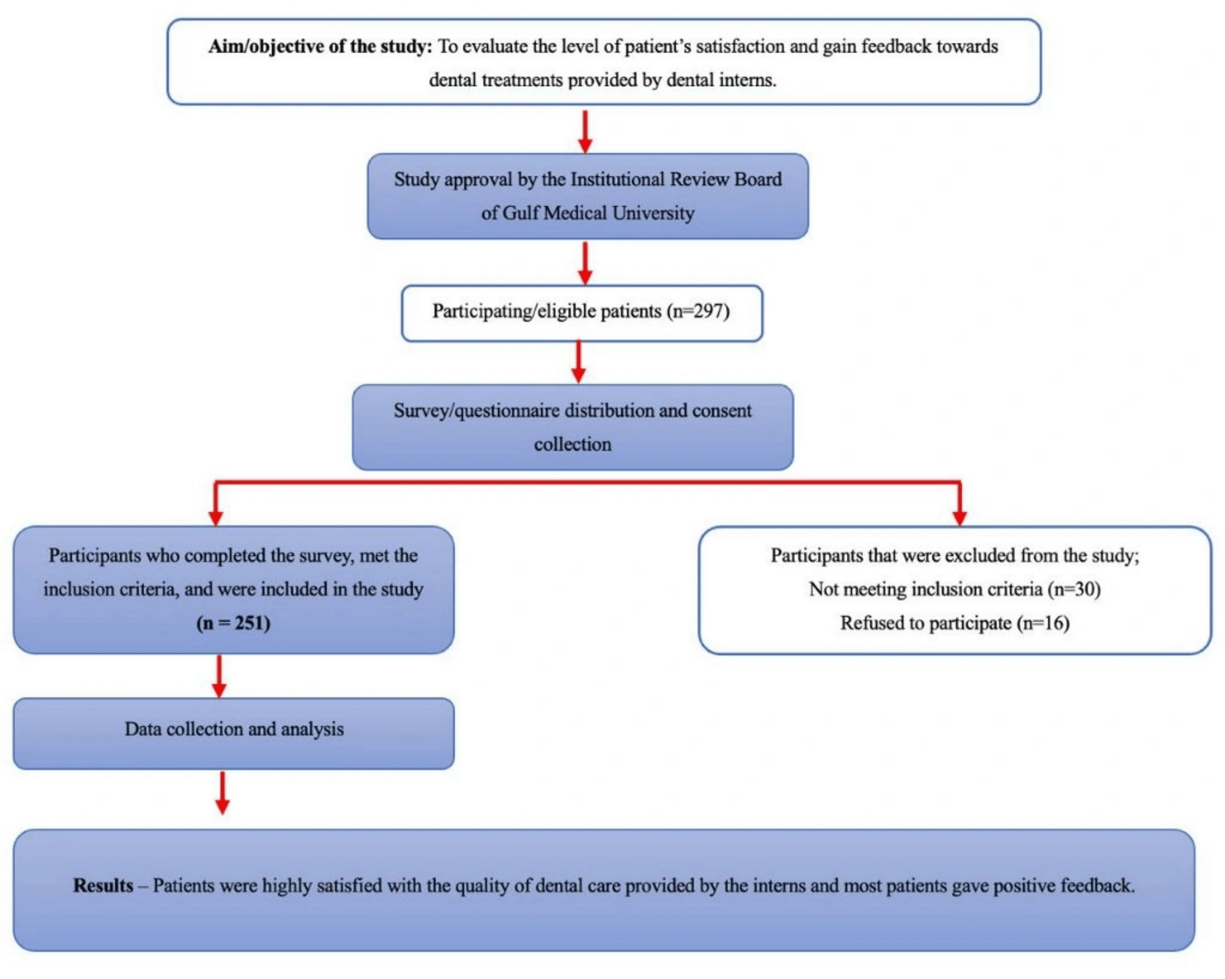
The study design and results

This cross-sectional, population-based survey is made to assess patient satisfaction with dental treatments provided by dental interns through survey distribution. Figure 1 illustrates the study design.

Sample size estimation

The sample size was calculated using the following formula: n₀ = (Z² pq)/e², where e is the desired level of precision (i.e., the margin of error), p is the estimated proportion of the population with the attribute in question, and q is equal to 1-p. For this study, a 95% CI with a precision of ±0.05 was selected. At a 95% CI, the Z value is 1.96 (as per standard normal distribution tables). Substituting the values into the formula gives: n₀=(1.96)²×(0.5)×(0.5)/(0.05)²=385.

However, since the population under study, patients treated by interns at the TDH, is relatively small, the sample size was adjusted using the finite population correction formula: n=n₀/(1+((n₀−1)/N)), where n₀ is the sample size recommended by Cochran’s formula, and N is the population size. Given an estimated population of approximately 700 patients, the adjusted sample size is calculated as: n=385/(1+(384/700))=250.

Inclusion criteria

The study participants were selected by the convenience method from the outpatient dental training hospital, TDTH, Ajman, UAE, within the academic year 2023-2024. The participants were informed by the treating dental interns to read the informed consent and accept it if they wished to participate, before proceeding to answer the questionnaire. The study population included any patient from the age of 15 and above, who is able to read and understand the questionnaire, and is treated by dental interns at TDTH. Informed consents for participants aged 15-17 years were obtained by a parent or a legal guardian prior to participation. 

Exclusion criteria

The exclusion criteria included patients who cannot read and understand the questionnaire, patients that refused to participate in the study, patients who are not getting treated by dental interns, patients under the age of 15, and special needs patients.

Survey methodology and implementation

The questionnaire was designed in the English language and translated into Arabic. The translated version was revised by the researchers, all of whom understood and were fluent in the Arabic language, to ensure that the meaning of the questions remained unchanged. The study's questionnaire was modified from a previously approved questionnaire that evaluated patient satisfaction in several important areas. The original form and objective of the questions were preserved, but minor adjustments were made to better suit our clinical context. Although the adapted version was not re-validated, the original tool has established validity, and the adapted version was reviewed for clarity and relevance prior to use [12]. A pilot study was conducted on the first 15 patients to ensure that the method of data collection and the questions were valid for the study. The questionnaire was easily understood based on patients’ feedback and was finalized accordingly.

The self-administered questionnaire was distributed to the eligible patients by the researchers. The targeted population was surveyed using a "Google Forms" questionnaire, which was created to collect participants’ informed consent and responses. The questionnaire was distributed to patients either by sending them the link or by allowing them to scan a generated QR code to complete it on-site. If any participant needed any help or had any trouble understanding the questions due to language barriers, the researchers assisted in the translation of the questions.

The questionnaire was divided into two sections consisting of 11 questions (nine closed-ended and two open-ended questions). The first section includes socio-demographic questions (gender and age). The second section consists of questions regarding the patient’s chief complaint, the type of treatment the patient received, pain assessment, and their satisfaction level. The pain was assessed using the Visual Analog Scale (VAS), ranging from 0 to 10, where 0 indicates no pain and 10 indicates the highest level of pain. The patient satisfaction level was evaluated using a "4-point Likert Scale" ranging from very satisfied (4) to very dissatisfied (1) [1]. It also included questions to assess their satisfaction with the treating doctor, their ability to manage difficult situations, and their satisfaction with the dental center’s services.

Statistical analysis and data collection

The data analysis was conducted using the IBM SPSS Statistics Version 29.0.2.0 (20) (IBM Corp., Armonk, NY) and analyzed using descriptive statistics. Frequencies, mean, and standard deviation (SD) were calculated for qualitative and quantitative data. A Chi-square test was used to compare observed values between two variables such as patient satisfaction and pain. The level of significance was set at p<0.05 with a 95% CI.

## Results

A total of 251 patients participated in the questionnaire, of whom 150 (59.8%) were males and 101 (40.2%) were females. Patients ranged in age from 15 and over 55 (Table 1). The main reason patients visited the dental hospital was pain (33.9%) (Table 2). Patients measured their pain levels preoperatively and postoperatively using the VAS, ranging from 0 to 10, with 0 representing no or the lowest pain and 10 representing the highest pain level. Most patients experienced mild to severe pain (66.7%) before their treatment was initiated. The overall pain level decreased immediately post-treatment, with approximately 12% of patients experiencing mild residual pain and 6.8% experiencing moderate to severe pain (Table 3). Patients seeking pain relief were significantly more satisfied (66.1%) than those who came for routine care (33.9%) (p<0.001) (Table 2).

**Table 1:** Association between dental satisfaction scores, sociodemographic characteristics, and patient's chief complaints A mean value between 1.00 and 2.00 suggests that most patients fall into the "very satisfied" or "satisfied" categories. SD, standard deviation

Characteristics		No (n=251)	%	Mean satisfaction score and SD
Gender	Male	150	59.8	1.18 (0.435)
	Female	101	40.2	1.15 (0.384)
Age	15-20 yrs	31	12.4	1.32 (0.64)
	21-30 yrs	67	26.7	1.15 (0.40)
	31-45 yrs	101	40.2	1.09 (0.29)
	46-55 yrs	29	11.6	1.28 (0.45)
	>55 yrs	23	9.2	1.22 (0.41)
Chief complaint	Checkup	45	17.9	1.11 (0.38)
	Pain	85	33.9	1.11 (0.31)
	Tooth replacement	25	10.0	1.16 (0.37)
	Cleaning	19	7.6	1.05 (0.22)
	Filling	30	12.0	1.13 (0.34)
	Tooth removal	18	7.2	1.22 (0.45)
	Root canal	9	3.6	1.33 (0.47)
	Other (suture removal, temporary restoration, and fluoride and sealant placement)	20	8.0	1.55 (0.74)

**Table 2:** Patient satisfaction based on the initial presence of pain p<0.001; chi-square analysis SD, standard deviation

Presence of pain	No (n=251)	%	Mean satisfaction score and SD
No	85	33.9	1.22 (0.49)
Yes	166	66.1	1.14 (0.36)

**Table 3:** Frequency of preoperative and postoperative pain scale scores among participants

Pain scale		No (n=251)	%
Preoperative pain	Mild (1-3)	28	11.2
	Moderate (4-6)	52	20.8
	Sever (7-10)	87	34.7
Postoperative pain	Mild (1-3)	31	12.4
	Moderate (4-6)	7	2.8
	Severe (7-10)	10	4

The most frequently performed dental treatment that was provided by the interns was composite restorations (82/295; 27.8%), followed by endodontic treatment (63/295; 21.4%), and (60/295; 20%) dental extractions. All the patients who received these types of treatments were satisfied with the outcome (Table 4).

**Table 4:** Dental satisfaction score by the type of treatment received The chi-square analysis reflects the distribution of treatments performed (p=0.002). Some patients received more than one treatment (n=44), while others received one type of treatment (n=207).

Treatment performed	Very satisfied		Satisfied		Dissatisfied		Very dissatisfied		Total
	Count	%	Count	%	Count	%	Count	%	n=295
Composite restorations	73	34.4	9	24.3	0	0.0	0	0.0	82 (27.8%)
Root canal treatment	51	24.1	12	32.4	0	0.0	0	0.0	63 (21.4%)
Extraction	50	23.6	10	27.0	0	0.0	0	0.0	60 (20.3%)
Scaling and polishing	41	19.3	4	10.8	0	0.0	0	0.0	45 (15.3%)
Dental prosthesis	30	14.2	5	13.5	0	0.0	1	100.0	36 (12.2%)
Other (suture removal, temporary restoration, and fluoride and sealant placement)	6	2.8	2	5.4	1	100.0	0	0.0	9 (3.1%)

Table 5 illustrates positive feedback from the patients who participated and their general satisfaction with dental care at TDTH. 72.1% reported that the intern was able to overcome clinical complications effectively (such as file separation and root separation during extraction procedures). Results indicate that most patients (99.2%) treated by dental interns at TDTH were significantly satisfied with the treatment they received, while only 0.8% were dissatisfied (p<0.001). Ultimately, a significant number of patients (99.6%) would visit and recommend TDTH to others after their full experience with dental care.

**Table 5:** Positive responses regarding the quality and satisfaction of dental care

Quality of care	Positive responses	%	Total responses
Dental intern professionalism	240	95.7	251
Intern's ability to handle complications according to patient’s perspective	31	72.1	43
Patients that will visit and recommend the hospital	250	99.6	251
General satisfaction with dental care received	249	99.2	251

## Discussion

The importance of our study lies in its assessment of patient satisfaction with dental services provided by interns, a key factor in ensuring the quality of care and maintaining the trust of patients in educational hospitals. By focusing on patient experiences at TDTH, this research contributes to the larger conversation about enhancing patient-centered care and refining dental education programs.

The results of Tashkandi et al.'s study and our study both indicate high levels of patient satisfaction in educational dental hospitals, with no significant differences in overall satisfaction scores among demographic groups. Their study showed the highest satisfaction with dentist professionalism (97.3%) and answering patients’ questions (97.3%), while the least satisfying factors were appointment scheduling (77.1%) and treatment completion efficiency (76.3%). Similarly, our study found strong satisfaction in communication and professionalism but identified challenges in appointment availability and treatment efficiency. Despite minor methodological differences, both studies highlight the need for improvements in scheduling and timely treatment completion [8].

In the study conducted by Abdulrahman Al Balhaddad, patient satisfaction in a dental institution in Saudi Arabia was 84% for appointment scheduling and 90% for facility satisfaction. In contrast, our study revealed an exceptionally high overall satisfaction rate of 99.2% (P=6.54×10⁻⁵⁴), with 100% of patients who received treatment reporting satisfaction and 99.6% willing to revisit and recommend the hospital. A unique aspect of our study was its assessment of pain relief as a factor influencing satisfaction, showing that patients seeking pain relief were significantly more satisfied (66.1%) than those receiving routine care (33.9%). Additionally, 72.1% of patients acknowledged interns’ ability to manage clinical complications effectively [9].

In a study by Klaassen et al., patient satisfaction in a university dental clinic was influenced by four main factors: emotional care, skills and treatment, patient expectations, and communication. Their findings highlight the importance of empathy and clear communication in enhancing the patient experience. In comparison, our study at TDTH found that satisfaction was primarily affected by clinical outcomes, such as pain relief, treatment success, and the ability of interns to manage complications. It is possible that this is due to the lack of assessment questions regarding emotional care and communication in our survey. This difference may be attributed to variations in patient demographics, expectations, and the nature of care provided in each setting [10].

In comparison to the study by Leow and Liew (2022), which found that longer consultation times and better communication between healthcare providers and patients were positively associated with higher satisfaction levels, our study did not specifically assess these factors. Leow and Liew’s research emphasized that clear communication and empathy were key drivers of patient satisfaction, particularly in primary care settings, where patient-provider interaction is important. In contrast, our study focused primarily on treatment outcomes and found that patients expressed high satisfaction with specific dental procedures, such as composite restorations, endodontic treatments, and extractions. One notable difference is that while our study found satisfaction levels to be more related to clinical outcomes, Leow and Liew observed a variation in satisfaction based on consultation time and patient demographics [11].

In comparing the results of our study with Hashim’s (2005) research on patient satisfaction with dental services at Ajman University, several similarities and differences arise. Both studies show high levels of satisfaction among patients, with our study reporting an impressive 99.2% satisfaction rate, which is closely aligned with Hashim’s finding of 93.5% overall satisfaction. Both studies also highlight pain as a significant factor affecting patient satisfaction, with a substantial portion of patients in our study (66.7%) reporting mild to severe pain pre-treatment. After treatment, most patients in our study experienced a decrease in pain, with only 12% reporting mild residual pain and 6.8% reporting moderate to severe pain. Hashim’s study did not explicitly discuss pain levels, but his research indicated that factors such as the quality of treatment and effective communication significantly impacted overall satisfaction [1].

Future research in this area could benefit from larger sample sizes to improve the final findings. Expanding the distribution for the survey, perhaps by using digital platforms or employing additional personnel, could enhance response rates. Furthermore, conducting longitudinal studies to track changes in patient satisfaction over time or across different educational hospitals may provide deeper insights. Finally, incorporating other methods like focusing more on empathy and emotional care for survey questions would further ensure neutrality and reliability, strengthening the results and making them more comprehensive.

Finally, our study encountered certain limitations. The sample size of 251 participants may not comprehensively show the diversity of patient experiences. Additionally, the distribution of the survey had some challenges, as only a few individuals were available to contribute to distributing the surveys, potentially restricting the number of participants. The sampling technique used in the research was convenience sampling, which may limit the generalizability of the findings. Another limitation is the potential for response bias or a ceiling effect, as the overall satisfaction rate was very high (99.2%). This may be due to social desirability bias or participants’ reluctance to provide critical feedback, particularly in the context of being surveyed by their treating interns. Despite these challenges, the survey questions were carefully designed to minimize bias and provide an accurate assessment of patient satisfaction.

## Conclusions

Most patients at TDTH were significantly satisfied with the quality of the treatments and services provided by the dental interns. The findings highlight the effectiveness of the hospital's training program in preparing skilled dental professionals. Additionally, the results suggest that providing high standards in supervision and mentorship plays an important role in gaining patients trust and improving their satisfaction. The continuous monitoring and enhancement of patient satisfaction will ensure continued excellence in dental care delivery.

## Appendices

Title: Patient Satisfaction with Dental Services Performed by Dental Interns at Thumbay Dental Hospital, Ajman

Investigator(s): Lara Kanjee, Marwa Al Hadi, Noor Alyasri Supervisor: Dr. Bassem Eid Site(s): Thumbay Dental Hospital, Ajman Study-Related Phone Number(s): 0501219622, 0582425547, 0502025653

This consent form gives you information about the study we wish to conduct. You will get to know the benefits you may get if you agree to participate, how we hope to use the information that we will get from the study, and who else will share the information. This will help you to make an informed decision whether to participate or not.

You may choose not to participate, or you may withdraw your decision to participate in the study at any time. In either case, you will not lose any benefits to which you are otherwise entitled.

What is the purpose of this study?

To evaluate the level of patient satisfaction and needs for dental treatments provided by dental interns in the years 2023-2024 at Thumbay Dental Training Hospital, Ajman, and to gain feedback on these services.

Why am I being asked to participate in this study?

You are asked to participate in this research because you fulfill the inclusion criteria.

How long will I be in this study?

You will be in this study for 10-15 minutes that are needed for answering a questionnaire.

How many other subjects will be participating in this study?

The study will include Thumbay Dental Hospital patients treated by dental interns during their practice 2023-2024 who will be selected randomly.

What will I be asked to do?

You will be asked to fill a questionnaire that involves your experience and feedback after being treated by dental interns at Thumbay Dental Hospital.

What benefits can I expect from participating in the study?

Participation in the study will help the investigators collect data about patient's experience and satisfaction at Thumbay Dental Training Hospital.

Assurance of Confidentiality, Anonymity, and Storage of Data:

We assure you that the study is anonymous. Confidentiality of the information will be respected and only the research team, faculty members, statistician, and Institutional Review Board member may have access to the data.

Provision of Feedback to the Participant:

The results of this study will be published/presented at national/international conferences.

Can I participate in this study if I do not sign this consent form?

To participate in this research, you have to sign this informed consent, then you will be provided with a copy.

Consent

I have read this consent form. All my questions have been answered. I volunteer to take part in this study. I authorize the use and disclosure of my questionnaire’s responses to the parties listed in the authorization section of this consent for the purposes described above.

Signature of Subject: _______________________________________             Date: ___________

Signature of the Principal Investigator: ___________________________          Date: ___________

Questions

Section 1:

Social-Demographic Characteristics:

Gender:

☐ Male ☐ Female

Age:

☐ 15-20 ☐ 21-30 ☐ 31-45 ☐ 46-55 ☐ 55 or above

Section 2:

What was your chief complaint/reason that made you visit the dental hospital? ……………………………………………………………………………

Did you experience any pain before visiting the dental hospital? ☐ Yes ☐ No

Please rate your pain before treatment: ……………………………………………………………………………

Which of the following treatments did you receive? (You can select more than one option) ☐ Filling ☐ Root canal ☐ Cleaning ☐ Teeth replacement (crown/bridge/denture) ☐ Tooth removal (extraction) ☐ Other dental treatment: ……………………………………………………………

How would you describe your satisfaction level after completion of the treatment? ☐ Very satisfied ☐ Satisfied ☐ Dissatisfied ☐ Very dissatisfied

Please rate your pain after treatment: ……………………………………………………………………………

How would you rate the professionalism of your treating dentist/intern? ☐ Poor ☐ Fair ☐ Good ☐ Excellent

Did your dentist/intern explain your treatment plan in detail? ☐ Yes ☐ No

Did you face any issues regarding your dental treatment? If yes, rate how it was handled. (If not, skip to the next question.) Poor ☐ ☐ ☐ ☐ ☐ Excellent

Would you visit again and/or recommend Thumbay Dental Hospital to your family and friends? ☐ Yes ☐ No

Do you have any suggestions for improvement? ……………………………………………………………………………
